# Relationships between Pre-Stroke SARC-F Scores, Disability, and Risk of Malnutrition and Functional Outcomes after Stroke—A Prospective Cohort Study

**DOI:** 10.3390/nu13103586

**Published:** 2021-10-13

**Authors:** Masafumi Nozoe, Hiroki Kubo, Masashi Kanai, Miho Yamamoto

**Affiliations:** 1 Department of Physical Therapy, Faculty of Nursing and Rehabilitation, Konan Women’s University, Kobe 658-0001, Japan; kanaimasa07@gmail.com; 2 Department of Rehabilitation, Itami Kousei Neurosurgical Hospital, Itami 664-0028, Japan; hiro.k16862@gmail.com (H.K.); bf39my23@gmail.com (M.Y.)

**Keywords:** stroke, sarcopenia, SARC-F score, disability, malnutrition risks

## Abstract

SARC-F is a screening tool for sarcopenia; however, it has not yet been established whether SARC-F scores predict functional outcomes. Therefore, we herein investigated the relationship between SARC-F scores and functional outcomes in stroke patients. The primary outcome in the present study was the modified Rankin Scale (mRS) 3 months after stroke. The relationship between SARC-F scores and poor functional outcomes was examined using a logistic regression analysis. Furthermore, the applicability of SARC-F scores to the assessment of poor functional outcomes was analyzed based on the area under the receiver operating curve (ROC). Eighty-one out of the 324 patients enrolled in the present study (25%) had poor functional outcomes (mRS ≥ 4). The results of the multivariate analysis revealed a correlation between SARC-F scores (OR = 1.29, 95% CI = 1.05–1.59, *p* = 0.02) and poor functional outcomes. A cut-off SARC-F score ≥ 4 had low-to-moderate sensitivity (47.4%) and high specificity (87.3%). The present results suggest that the measurement of pre-stroke SARC-F scores is useful for predicting the outcomes of stroke patients.

## 1. Introduction

Stroke is one of the leading causes of disability [1], and the primary cause of disability in stroke patients is brain injury [2]. Stroke increases the risk of sarcopenia [3], which is a contributing factor to disability in these patients [4]. The prevalence of stroke-related sarcopenia increases in the elderly and is associated with the poor recovery of activities of daily living [5,6]. Although sarcopenia may develop in stroke patients, it may also be present pre-stroke, and has been implicated in poor functional outcomes [7,8,9].

SARC-F is a screening tool for sarcopenia in elderly subjects [9,10,11,12] and predicts the risk of pre-stroke sarcopenia [9]. A previous study reported that the risk of developing sarcopenia was elevated in patients with a SARC-F score ≥4 [13]. Since SARC-F has low sensitivity, but high specificity [11,14], the risk of sarcopenia assessed by SARC-F may be underestimated [11,14,15,16]. Another cut-off score was recently reported for predicting the risk of sarcopenia (SARC-F = 1) [17] and the usefulness of SARC-F for detecting the risk of frailty or falls has been demonstrated [18,19]. A previous study revealed a relationship between SARC-F scores and muscle mass [20,21]. Based on these findings, SARC-F scores may be useful for predicting the risk of pre-stroke sarcopenia and functional outcomes in stroke patients.

The aim of the present study was to investigate the relationship between pre-stroke SARC-F scores and functional outcomes in stroke patients. We hypothesized that SARC-F scores are associated with functional outcomes, even after adjustments for confounding factors, such as pre-stroke disability or a risk of malnutrition.

## 2. Materials and Methods

### 2.1. Study Design and Participants

This prospective cohort study was conducted between August 2017 and December 2019 and included elderly patients consecutively admitted to Itami Kousei Neurosurgical Hospital within 48 h of stroke onset. The following patients were enrolled: age ≥ 65 years and evidence of cerebral infarction or intracerebral hemorrhage on computed tomography or magnetic resonance imaging. Exclusion criteria were (1) pre-stroke dependent ambulation, (2) patients unable to complete the questionnaire because of impaired consciousness, cognitive dysfunction, or language disorders, such as aphasia, and (3) the lack of informed consent. The present study was approved by the Research Ethics Committee of Konan Women’s University, and all patients provided their informed consent.

### 2.2. SARC-F

The SARC-F questionnaire, which evaluates the pre-stroke status and has been adapted for a Japanese population, was completed by patients within 5 days of admission. It comprises the following components: strength, assistance with walking, rising from a chair, climbing stairs, and falls [12]. SARC-F scores range between 0 and 10 (0 = best, 10 = worst), with each component receiving 0–2 points.

### 2.3. Assessment of a Risk of Malnutrition and Comorbidities

The Geriatric Nutritional Risk Index (GNRI) was employed to evaluate the nutritional status of patients [22], and was calculated as follows: GNRI = (1.489 × serum albumin [g/dL]) + 103 (41.7 × weight [kg]/ideal body weight). In cases in which weight/ideal body weight was ≥1.0, the ratio was set to 1. GNRI ≤ 98 kg/m^2^ was defined as a risk of malnutrition as previously reported [22].

### 2.4. Clinical Characteristics

Information was obtained from electronic medical records on the following patient characteristics: age, sex, height, body weight, body mass index, neurological deficits evaluated by the National Institutes of Health Stroke Scale (NIHSS) score, stroke type, lesion laterality, and pre-stroke mRS. Pre-stroke disability was defined as mRS = 2 (slight disability) or 3 (moderate disability), and no disability as mRS = 0 (no symptoms) or 1 (no significant disability).

### 2.5. Main Outcome

The primary outcome of the present study was the modified Rankin Scale (mRS) evaluated 3 months after stroke from medical records or in a telephone interview. mRS was scored based on an unstructured direct interview by a physician [23], with 0 = no symptoms, 1 = no significant disability despite the presence of symptoms; capable of performing all of the usual duties and activities, 2 = slight disability; unable to perform all of the previous activities, but capable of attending to one’s own affairs without assistance, 3 = moderate disability; requiring some help, but capable of walking without assistance, 4 = moderately severe disability; unable to walk or attend to one’s own bodily needs without assistance, 5 = severe disability; bedridden, incontinent, and requiring constant nursing care and attention, and 6 = dead [24]. A poor outcome was defined as mRS scores 3 months after stroke of 4–6 [25].

### 2.6. Statistical Analysis

Data are shown as medians (interquartile range; IQR) and numbers (%) for categorical data. The unadjusted and adjusted odds ratios (OR) of SARC-F scores and poor functional outcomes (mRS ≥ 4) were calculated by a logistic regression analysis. Confounding factors were adjusted for, including age [26], sex [27], NIHSS [28], pre-stroke disability [29], and malnutrition risk [22,30]. The applicability of SARC-F scores to evaluations of poor functional outcomes was examined in an analysis of the area under the receiver operating curve (ROC). Sensitivity and 1-specificity were calculated from the obtained sensitivity and specificity, and the point at the maximal value was taken as the optimum cut-off value. The area under the curve, sensitivity, specificity, and positive and negative predictive values for SARC-F were analyzed. All statistical analyses were performed using SPSS version 20.0 (SPSS, Inc., Chicago, IL, USA). Differences with *p* < 0.05 were considered to be significant.

## 3. Results

During the study period, 643 elderly stroke patients were hospitalized, and 293 were excluded from the analysis due to admission 48 h after stroke symptom onset (*n* = 17), pre-stroke dependent ambulation (*n* = 74), impaired consciousness (*n* = 68), cognitive dysfunction (*n* = 64), and aphasia (*n* = 39). Ten patients also refused to participate and 21 did not provide informed consent. Among the 350 patients enrolled, 26 patients refused a follow-up. Therefore, 324 stroke patients were included in the present study.

Among the patients enrolled, 54 (17%) had pre-stroke disability and 41 (13%) were at risk of malnutrition. Eighty-one patients (25%) had poor functional outcomes (mRS ≥ 4) 3 months after stroke (Table 1). Table 2 shows the distribution for each SARC-F score in all patients. Approximately 50% of patients had an SARC-F score of zero.

Based on unadjusted OR, age (OR = 1.06, 95% confidence interval (CI) = 1.02–1.10, *p* = 0.007), NIHSS (OR = 1.40, 95% CI = 1.36–1.68, *p* < 0.001), pre-stroke disability (OR = 5.75, 95% CI = 3.00–10.99, *p* < 0.001), risk of malnutrition (OR = 3.78, 95% CI = 1.86–7.68, *p* < 0.001), and SARC-F scores (OR = 1.44, 95% CI = 1.27–1.64, *p* < 0.001) correlated with poor functional outcomes 3 months after stroke.

After adjustments for these factors, NIHSS (OR = 1.56, 95% CI = 1.39–1.76, *p* < 0.001), pre-stroke disability (OR = 3.22, 95% CI = 1.11–9.34, *p* = 0.03), and SARC-F scores (OR = 1.29, 95% CI = 1.05–1.59, *p* = 0.02) correlated with poor functional outcomes (Table 3).

**Figure 1:**
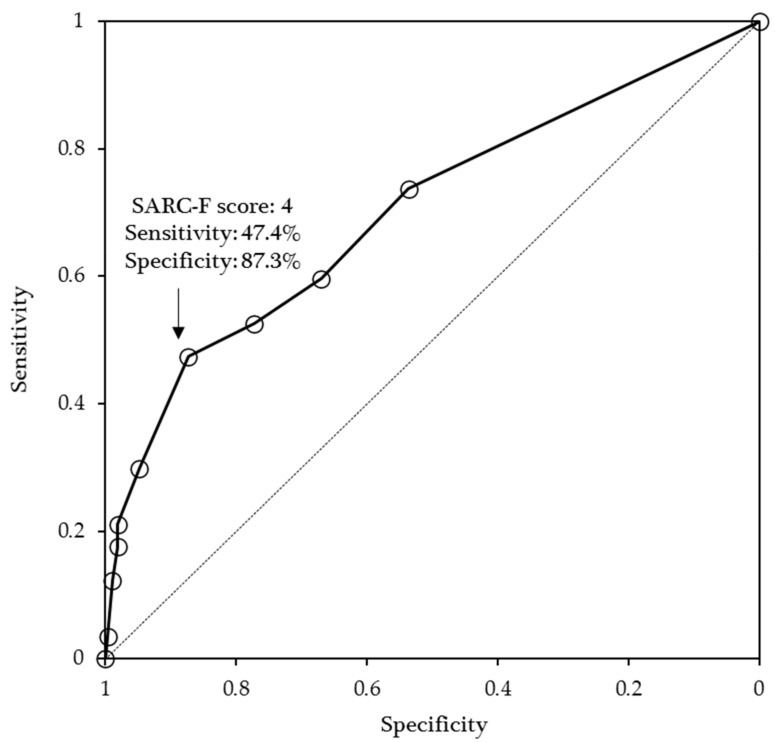
Receiver operating characteristic curves of SARC-F scores as an indicator of poor outcomes.

The results of the ROC curve analysis are shown in Figure 1. An SARC-F cut-off ≥4 had low to moderate sensitivity (47.4%) and high specificity (87.3%) to screen for poor functional outcomes (positive predictive value = 44.3%, negative predictive value = 88.6%, AUC = 0.702, 95% Cl = 0.620–0.784).

## 4. Discussion

This prospective cohort study examined the relationship between functional outcomes and pre-stroke SARC-F scores in stroke patients. The results obtained revealed the independent effects of pre-stroke SARC-F scores on functional outcomes and also supported the applicability of an SARC-F score ≥ 4 for predicting poor functional outcomes in stroke patients.

Previous studies reported a relationship between pre-stroke sarcopenia and poor outcomes [7,8,9]; however, these studies did not consider pre-stroke disability as a confounding factor. Sarcopenia is generally associated with disability [31,32,33], and pre-stroke disability has a negative impact on functional outcomes in stroke patients [29,34]. Therefore, the effects of pre-stroke sarcopenia on physical function need to be interpreted in consideration of pre-stroke disability. If a patient responds “unable”, the results of SARC-F also need to be considered as the result of an assessment of disability. However, the present results demonstrated the independent effects of SARC-F scores on poor functional outcomes even after adjustments for pre-stroke disability. Therefore, pre-stroke SARC-F scores are useful not only for sarcopenia screening, but also for predicting poor functional outcomes in stroke patients.

The cut-off SARC-F score for sarcopenia screening is ≥4; however, a previous study demonstrated the low sensitivity and high specificity of this score for detecting sarcopenia [11,14]. We also investigated whether a cut-off SARC-F score ≥ 4 had low to moderate sensitivity and high specificity for the screening of poor functional outcomes in stroke patients. A SARC-F score ≥ 4 was previously associated with poor functional outcomes; however, this cut-off value was not validated [9]. The present results confirmed the validity of a SARC-F score ≥ 4 in stroke patients.

There are a number of limitations that need to be addressed. Younger patients or those with consciousness disorders, severe cognitive dysfunction, or aphasia were excluded from the present study. Since severe stroke patients were not enrolled, the present results may not be applicable to all stroke patients. Furthermore, the present study was conducted in a small, single-center setting and did not adjust for many confounding factors. Another limitation is that the impact of SARC-F scores on mortality or long-term outcomes was not examined. Therefore, further multicenter, large-scale, and long-term studies are needed to obtain more general and useful results for these patients.

## 5. Conclusions

Pre-stroke SARC-F scores were associated with poor functional outcomes in stroke patients even after adjustments for confounding factors, such as pre-stroke disability or a risk of malnutrition, and a SARC-F score ≥ 4 was suitable for predicting poor outcomes in these patients. Assessments of the pre-stroke status using SARC-F scores may be useful for predicting the outcomes of stroke patients.

## Figures and Tables

**Table 1:** Stroke patient characteristics.

	Total Cohort
	(*n* = 324)
Age (years, median (IQR))	76 (11)
Sex (male/female)	187/137
Body mass index (kg/m^2^, median [IQR])	22.5 (4.2)
NIHSS (median [IQR])	2 (3)
Stroke type (infarction/hemorrhage)	267/57
Lesion side (right/left/both)	161/152/11
Pre-stroke disability (%)	54 (17)
Stroke risk factors (%)	
Hypertension	159 (49)
Diabetes	74 (23)
Previous stroke	89 (28)
Hypercholesterolemia	78 (24)
Ischemic heart disease	27 (8)
Atrial fibrillation	27 (8)
Smoking	110 (34)
Risk of malnutrition (%)	41 (13)
mRS 3 months after stroke (%)	
0	31 (10)
1	105 (32)
2	58 (18)
3	49 (15)
4	69 (21)
5	10 (3)
6	2 (1)
Poor functional outcome (%)	81 (25)

IQR = interquartile range; NIHSS = National Institutes of Health Stroke Scale; mRS = modified Rankin Scale.

**Table 2:** SARC-F scores and their distribution.

	Total Cohort
	(*n* = 324)
SARC-F score (median [IQR])	1 (3)
SARC-F score distribution	
score = 0 (%)	158 (49)
score = 1 (%)	44 (13)
score = 2 (%)	31 (10)
score = 3 (%)	30 (9)
score = 4 (%)	30 (9)
score = 5 (%)	14 (4)
score = 6 (%)	2 (1)
score = 7 (%)	5 (2)
score = 8 (%)	7 (2)
score = 9 (%)	3 (1)
score = 10 (%)	0 (0)

IQR = interquartile range.

**Table 3:** Logistic regression analysis of poor functional outcomes.

	Unadjusted		Adjusted	
	Odds Ratio	*p*-Value	Odds Ratio	*p*-Value
	(95% CI)		(95% CI)	
Age	1.06	0.007	1.03	0.41
	(1.02–1.10)		(0.97–1.09)	
Sex	1.4	0.25	0.73	0.46
	(0.79–2.48)		(0.31–1.69)	
NIHSS	1.51	<0.001	1.56	<0.001
	(1.36–1.68)		(1.39–1.76)	
Pre-stroke disability	5.75	<0.001	3.22	0.03
	(3.00–10.99)		(1.11–9.34)	
Risk of malnutrition	3.78	<0.001	2.5	0.08
	(1.86–7.68)		(0.89–7.06)	
SARC-F score	1.44	<0.001	1.29	0.02
	(1.27–1.64)		(1.05–1.59)	

CI = confidence interval; NIHSS = National Institutes of Health Stroke Scale.

## Data Availability

The data presented in this study are available upon request from the corresponding author following permission by the Ethics Committee and the hospital at which the study was conducted.
